# Comparison of Concordance between Chuna Manual Therapy Diagnostic Methods (Palpation, X-ray, Artificial Intelligence Program) in Lumbar Spine: An Exploratory, Cross-Sectional Clinical Study

**DOI:** 10.3390/diagnostics12112732

**Published:** 2022-11-08

**Authors:** Jin-Hyun Lee, Hyeonjun Woo, Jun-Su Jang, Joong Il Kim, Young Cheol Na, Kwang-Ryeol Kim, Eunbyul Cho, Jung-Han Lee, Tae-Yong Park

**Affiliations:** 1 Institute for Integrative Medicine, Catholic Kwandong University International St. Mary’s Hospital, 25 Simgok-ro 100 Beon-gil, Seo-gu, Incheon 22711, Republic of Korea; 2 Department of Korean Medicine Rehabilitation, College of Korean Medicine, Wonkwang University, 460 Iksan-daero, Iksan-si 54538, Republic of Korea; 3 Digital Health Research Division, Korea Institute of Oriental Medicine, 1672 Yuseong-daero, Yuseong-gu, Daejeon 34054, Republic of Korea; 4 Department of Neurosurgery, Catholic Kwandong University International St. Mary’s Hospital, Catholic Kwandong University College of Medicine, 25 Simgok-ro 100 Beon-gil, Seo-gu, Incheon 22711, Republic of Korea; 5 Department of Acupuncture and Moxibustion Medicine, College of Korean Medicine, Wonkwang University, 460 Iksan-daero, Iksan-si 54538, Republic of Korea

**Keywords:** artificial intelligence, Chuna manual therapy, diagnosis, manual medicine, radiography

## Abstract

Before Chuna manual therapy (CMT), a manual therapy applied in Korean medicine, CMT spinal diagnosis using palpation or X-ray is performed. However, studies on the inter-rater concordance of CMT diagnostic methods, concordance among diagnostic methods, and standard CMT diagnostic methods are scarce. Moreover, no clinical studies have used artificial intelligence (AI) programs for X-ray image-based CMT diagnosis. Therefore, this study sought a feasible and standard CMT spinal diagnostic method and explored the clinical applicability of the CMT-AI program. One hundred participants were recruited, and the concordance within and among different diagnostic modalities was analyzed by dividing them into manual diagnosis (MD), X-ray image-based diagnosis (XRD) by experts and non-experts, and XRD using a CMT-AI program by non-experts. Regarding intra-group concordance, XRD by experts showed the highest concordance (used as a gold standard when comparing inter-group concordance), followed by XRD using the AI program, XRD by non-experts, and then MD. Comparing diagnostic results between the groups, concordance with the gold standard was the highest for XRD using the AI program, followed by XRD by non-experts, and MD. Therefore, XRD is a more reasonable CMT diagnostic method than MD. Furthermore, the clinical applicability of the CMT-AI program is high.

## 1. Introduction

Chuna manual therapy (CMT) is a manual therapy in Korean medicine in which Korean medicine doctors (KMDs) use hands, body parts, or assistive devices to stimulate the patient’s body to treat structural or functional problems [1]. Recently, manual therapy has been widely used as complementary alternative medicine to manage musculoskeletal pain [2]. Many studies have shown the efficacy of CMT [3,4,5,6,7]. Consequently, CMT was included in the coverage of the National Health Insurance of Korea in 2019. Therefore, interest in more accurate CMT diagnosis has increased.

CMT is administered to patients with functional imbalances and malpositions of the neuromuscular and musculoskeletal systems and aims to maintain homeostasis by simultaneously controlling the somatic, visceral, and mental systems [8]. For this, CMT spinal diagnosis is required. During diagnosis, the condition of the upper vertebral body is primarily identified relative to that of the lower vertebral body. The treatment site, technique, and direction are determined after diagnosis using manual methods by integrating the static perspectives of positional abnormality and the dynamic perspectives of joint motion restriction [9]. However, several studies have reported problems associated with poor validity and reliability of these manual methods [10,11,12,13,14]. Therefore, X-ray images and ultrasound examinations have recently been used to overcome the limitations of manual diagnostic methods and enhance their objectivity [15,16]. A study on CMT spinal diagnosis using X-ray imaging was also conducted [17]; however, it presented only the diagnostic criteria. Therefore, studies on the validity and reliability of CMT diagnosis by imaging are required.

Artificial intelligence (AI) has been used recently to identify spinal alignment [18]. For instance, in one study, AI was used to diagnose adult spinal deformity (ASD) and predict the consequences of ASD correction surgery [19]. Additionally, some studies have identified spinal curvature with AI using machine learning techniques [20,21]. Several studies have also focused on using AI for spine diagnosis. For example, in some studies, AI automatically recognized spinal markings through deep learning and analyzed alignment to aid diagnosis [22,23]. However, these studies have been limited to the field of conventional medicine for the identification of problems in specific areas, such as bone density [24] and spinal disc [25]; determining the need for surgery by predicting the degree of disease progression and postoperative results [26]; measurement of angle in scoliosis [18]; and analysis of stability in spondylolisthesis [27]. Furthermore, an X-ray image-based AI program that can be used in CMT diagnosis has been developed [28]. However, limited studies have investigated its diagnostic benefits through application in actual clinical practice. Moreover, studies analyzing intra- and inter-rater reliability and clinical diagnostic concordance of manual diagnosis (MD), X-ray image-based diagnosis (XRD), and diagnosis using an AI program are lacking.

The present study was performed to analyze the concordance within and among different diagnostic modalities by dividing the modalities into MD, XRD by experts, XRD by non-experts, and XRD using the CMT-AI program by non-experts after identifying CMT experts and non-experts. The aim of the study was to find a reasonable and standard CMT spinal diagnostic method with high diagnostic concordance and explore the clinical applicability regarding the diagnosis concordance and accuracy of the CMT-AI program.

## 2. Materials and Methods

### 2.1. Ethics and Trial Registration

This clinical study was approved by the institutional review board (IRB) of Wonkwang University Korean Medicine Hospital (IRB No. WKUIOMH-IRB-2021-08). The study protocol was registered in the Clinical Research Information Service, a clinical trials registry platform of the Disease Control and Prevention Agency under the South Korean Ministry of Welfare and Health [29]. The protocol for this clinical study has been published previously [30].

### 2.2. CMT Spinal Diagnostic Panel

CMT spinal diagnosis was performed by three CMT experts who performed MD, three CMT experts who performed XRD, and three CMT non-experts who performed XRD and XRD using a CMT-AI program. CMT experts were KMDs who fit one or more of the following descriptors while performing CMT in clinical practice: medical specialists of rehabilitation medicine of Korean medicine, Korean Society of Chuna Manual Medicine for Spine and Nerves (KSCMM) standard curriculum instructors, members of the board of education who had completed KSCMM’s regular workshop courses, or those who had published a paper related to Chuna medical imaging of the spine. CMT non-experts were KMDs who performed CMT in clinical practice but did not meet the criteria of a CMT expert.

### 2.3. Eligibility Criteria

#### 2.3.1. Inclusion Criteria

(a) Adults aged 20–60 years
(b) Patients who agreed to the clinical study plan and voluntarily signed the consent form approved by the IRB
(c) Patients who could communicate during physical examination and X-ray imaging

#### 2.3.2. Exclusion Criteria

(a) History of injury or surgery that might cause structural problems in the lumbar spine
(b) History of diseases that might cause deformation of the lumbar spine structure
(c) Difficulty in palpation of the spine due to moderate or higher obesity (>30 kg/m^2^) based on body mass index (BMI)
(d) Patients with psychotic disorders, alcoholism, or drug addiction
(e) Women who were pregnant or were likely to become pregnant
(f) Patients who were considered inappropriate to participate in this study according to the judgment of the principal investigator

### 2.4. Study Design

Study participants made the voluntary decision to participate in the study after the study protocol was explained to them in detail by a sub-investigator. They gave written informed consent on the first visit. After confirming that participants were not involved in other clinical trials, demographic data, including date of birth, age, sex, weight, height, and BMI, were collected. Vital signs, such as blood pressure, pulse, and body temperature, were determined for the eligible participants. The participants were asked to remain stable and not indulge in any sudden movements for 5 min prior to the measurement of vital signs.

Registration numbers were assigned to the study participants who were considered suitable for clinical study according to the inclusion and exclusion criteria, and vital sign and basic physical examinations were performed. Thereafter, three CMT experts independently performed MD on the participants (MD group), and L-spine standing anteroposterior and lateral view images of the study participants were obtained. MD and X-ray imaging were performed on the same day to minimize the variation in the position of the patient’s spine due to difference in measurement time.

**Figure 1:**
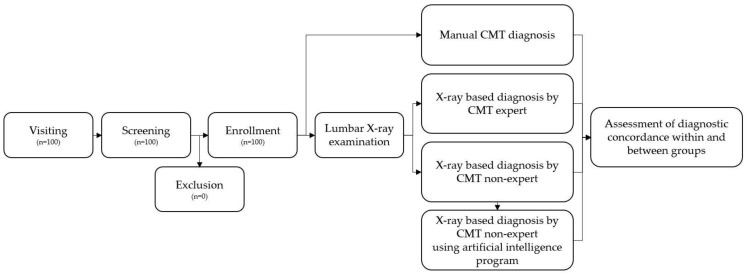
Flow chart of the study. CMT, Chuna manual therapy.

The remaining three CMT experts (XE group) and three CMT non-experts (XN group) independently performed CMT spinal diagnoses based on anonymized DICOM files. Approximately 1 month later, the three CMT non-experts re-executed XRD with the help of an AI program for the same cases (AI group). For each diagnostic method, we prepared standard operating procedures (SOPs), which were based on the Chuna Medical Textbook, a common textbook used by Colleges of Korean Medicine across Korea [8]; related previous studies [17,31]; and reference books related to manual medicine [32]. The researchers diagnosed the participants according to these SOPs. Based on the acquired diagnostic data on the spine, diagnostic concordance within and among groups was compared and analyzed (Figure 1).

Screening was performed by distinct researchers who were not diagnostic raters for the spine to avoid bias in evaluation. The raters who participated in MD performed the test in separate spaces. In addition, conversations among the raters were forbidden before and after diagnosis to prevent any chances of influence. In addition, X-ray image files were provided after anonymization, and diagnosis results were not shared.

### 2.5. Sample Size Calculation

This study was an exploratory clinical study. According to the Korean Ministry of Food and Drug Safety’s Regulations on Approval of Medical Device Clinical Trial Plan [33] and the Korean National Institute of Food and Drug Safety Evaluation Guidelines [34], sample size can be suggested by citing related prior research and supporting data if it is difficult to calculate the sample size by a statistical method. Therefore, a sample size of 100 participants was determined based on previous studies [18,35,36] related to the manual medicine diagnosis used in this study. Protocol details have been previously described [30].

### 2.6. CMT Diagnostic Methods in This Study

#### 2.6.1. CMT Spinal Diagnostic System

**Figure 2:**
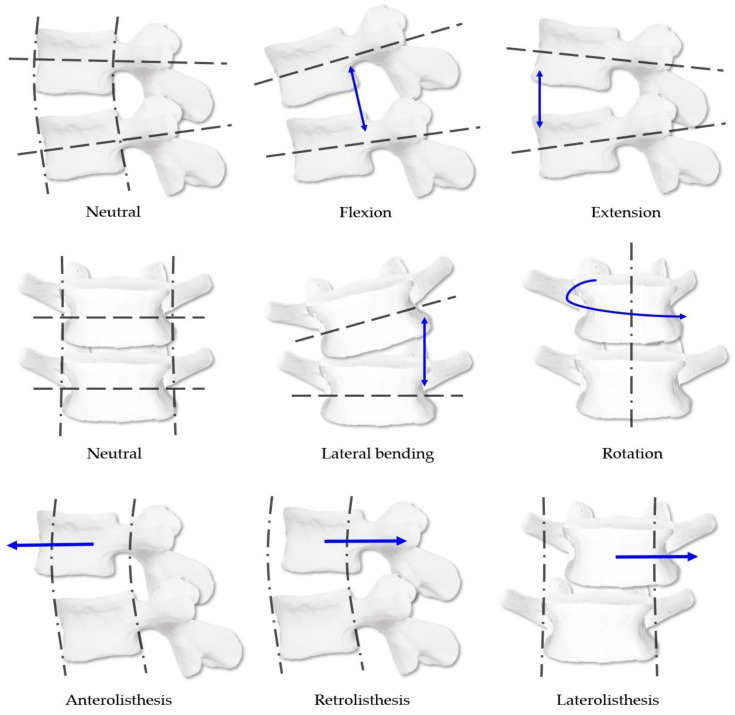
Classification system of spinal malposition and listhesis diagnosis in Chuna manual therapy. The blue arrow indicates how it changes from the neutral position according to the types of malposition or listhesis. Copyright 2022. Jin-Hyun Lee all right reserved.

CMT spinal diagnosis is based on three-dimensional movements. The standard kinesiologic system based on vertebral body malposition and limitations of joint range of motion is followed during diagnosis. Furthermore, the listing system of spinal malposition follows the Medicare method [8,9]. This study diagnosed malposition and listhesis of the upper vertebral body relative to the lower vertebral body for five lumbar spine levels. Figure 2 shows the detailed diagnosis system.

In the case of malposition, (1) investigators first checked for the presence of malposition. Malposition was considered present when the upper vertebral body relative to the lower vertebral body had any flexion, extension, rotation, or lateral bending malposition. Then, (2) the type of malposition in the sagittal plane (flexion, extension, or neutral), (3) in the axial plane (right rotation, left rotation, or neutral), and (4) in the coronal plane (right lateral bending, left lateral bending, or neutral) was identified. Only one diagnosis was possible within the items on each plane, and diagnoses (2–4) were considered independently of each other.

In the case of listhesis, (1) the presence of listhesis was first determined. Listhesis could be antero-listhesis, retro-listhesis, or laterolisthesis. At the time of diagnosis, investigators checked if the upper vertebral body was protruding relative to the lower vertebral body. Then, it was identified whether this corresponded to (2) anterolisthesis, retrolisthesis, or neutral, and (3) to right, left, or neutral laterolisthesis. Only one diagnosis was possible from options (2,3). Furthermore, diagnoses (2,3) were considered independent of each other.

#### 2.6.2. CMT Manual Diagnostic Method

**Figure 3:**
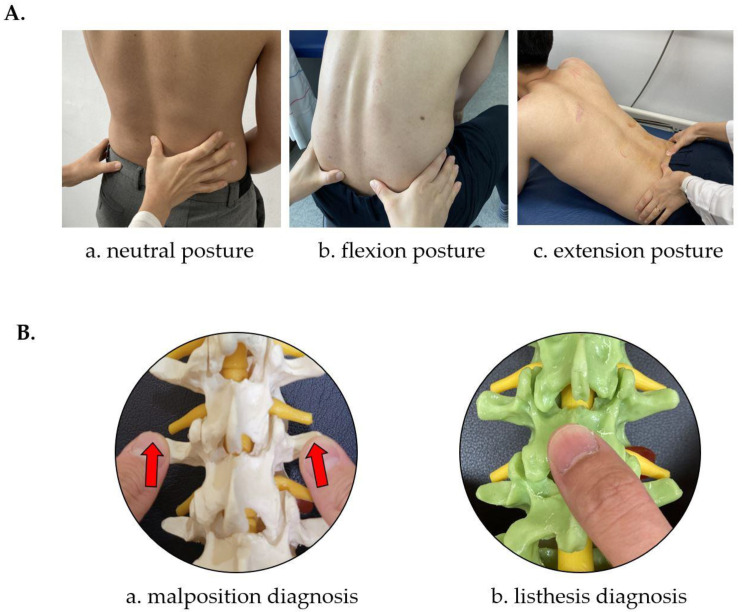
Practical example describing the actual diagnosis and palpation position. (**A**), posture for performing manual diagnosis in Chuna manual therapy; (**B**), contact point for diagnosis of malposition ((**a**) transverse process) and listhesis ((**b**) spinal process). Copyright 2022. Jin-Hyun Lee all right reserved.

When evaluating malposition using palpation, the study participants sat on a chair and maintained a neutral position with both feet on the floor. Raters checked the transverse processes of each lumbar segment. Then, the movement of the transverse processes was observed by asking the participants to flex or extend while palpating the transverse processes of each segment. (1) When a transverse process protruded more during the flexion posture and became symmetrical in the extension posture, extension, rotation, and lateral bending malposition were diagnosed. (2) When a transverse process protruded more in the extension posture and became symmetrical in the flexion posture, flexion, rotation, and lateral bending malposition were diagnosed. (3) If three or more transverse processes continued to protrude in the neutral, flexion, and extension postures, the corresponding segments were diagnosed as having neutral dysfunction in which lateral bending and rotation were in opposite directions. (4) A segment with no protrusion of transverse processes and no change in the space between the spinous processes during flexion and extension was evaluated as bilateral dysfunction. In addition, when the interval of a segment was narrower than that of the other segments, it was diagnosed as extension malposition, and when it was wider, it was diagnosed as flexion malposition (Figure 3 and Figure S1).

When evaluating listhesis using palpation, the study participants maintained a standing position with their feet shoulder-width apart, and raters checked the alignment of the lumbar spinous processes and the symmetry of the skin folds by examining their backs. Afterward, by palpating the spinous process along the midline of the patient’s spine, it was determined whether the spinous process was recessed or protruded, or if it deviated from the side on the vertical line connecting the spinous processes [37]. If the upper spinous process was concaved compared to the lower spinous process, it was diagnosed as anterolisthesis of the corresponding vertebral body. In contrast, if it was convexed, it was diagnosed as retrolisthesis. Laterolisthesis was diagnosed when the spinous processes were displaced from side to side in the midline arrangement of the spine, and other structures such as transverse processes or articular processes were deviated laterally compared to the same structures in the adjacent segments (Figure 3).

The test was completed when both malposition and listhesis were identified. The raters summarized their diagnosis in a case report form prepared in advance.

#### 2.6.3. CMT X-ray Image-Based Diagnostic Method

The raters followed the SOP and diagnosed the spine using the anonymized X-ray image data. The SOP proposed a diagnostic method based on the status of anatomical indicators (such as vertebral body, spinal process, intervertebral foramen, articular process, and intervertebral space) that appear according to the type of spinal malposition and listhesis [17]. Raters who diagnosed the conditions filled out case report forms in the same format as that used for spine diagnosis using palpation.

#### 2.6.4. CMT X-ray Image-Based Diagnostic Method Using the CMT-AI Program

**Figure 4:**
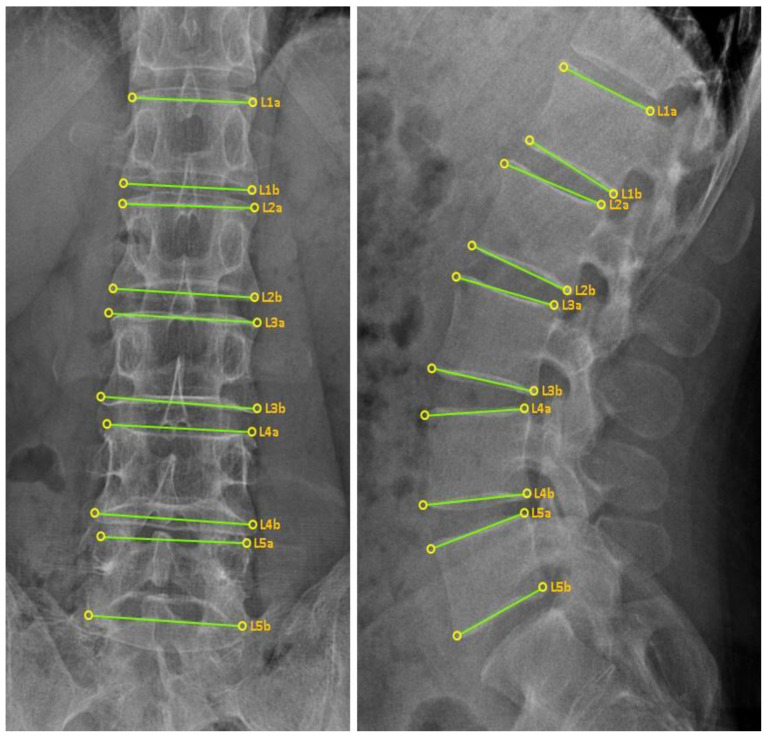
Example of the feature point locations suggested by the CMT-AI program. CMT, Chuna manual therapy; AI, artificial intelligence.

The CMT-AI program was a lumbar landmark detection program using a convolutional neural network developed for this study. The program automatically detects four outer vertices and the upper and lower surfaces of the vertebral body in a two-dimensional projected rectangular lumbar vertebral body image, which displays them as points and line segments. The program’s performance was good, with a detection success rate of 99.7% and a detection error of 4.54 ± 3.00 pixels [28]. Three CMT non-experts were provided with X-ray image data (Figure 4) containing information on the four vertices of the outer vertebral body. Then, the same cases were re-diagnosed using the CMT-AI program by the three non-experts. The results were recorded in the case report forms. An interval of 1 month from the end date of the first diagnosis was ensured to minimize the chance of investigators remembering the diagnosis performed based on image data only (without the aid of the AI program).

### 2.7. Study Outcomes

The intragroup diagnostic concordance of the three raters belonging to each group was compared. For comparison of concordance by diagnostic methods, all diagnostic results were divided into lumbar levels and detailed elements of the CMT spinal diagnostic system presented in Section 2.6.1.

In addition, raters who met the most qualification requirements for the expert group and raters with the highest level of SOP familiarity for the non-expert group were selected as representatives for each diagnostic method to compare concordance among diagnostic methods. Then, the diagnostic concordance of the four selected representatives was analyzed. Also, the diagnosis made by the representative of the XE group was used as a gold standard. Concordance between the gold standard and diagnostic results of representatives of the other groups was further analyzed individually.

### 2.8. Statistical Analysis

Descriptive statistics were used for the demographic data of the participants. Continuous data are expressed as a mean and standard deviation of the number of observations, and categorical data are expressed as frequency and percentage.

Kappa coefficients were used to analyze diagnostic concordance based on the collected data. Fleiss’ kappa coefficient was used to analyze the concordance of diagnosis results by the three raters in each group and the diagnostic concordance of the four representatives from each group [38]. Cohen’s kappa coefficient was used to analyze the diagnostic concordance between the gold standard and the representative raters of each group except that of the XE group [39]. Additionally, the criteria proposed by Landis and Koch [40] were used to confirm the degree of agreement with the derived kappa coefficient. Kappa value (κ) was identified as follows: <0, poor; 0.000–0.200, slight; 0.200–0.400, fair; 0.400–0.600, moderate; 0.600–0.800, substantial; and >0.800, almost perfect. Fleiss’ kappa coefficient analysis was performed using SAS^®^ (version 9.4, SAS Institute, Cary, NC, USA). Cohen’s kappa coefficient analysis was performed using SPSS statistics for Windows (version 22.0, IBM Co., Armonk, NY, USA).

## 3. Results

### 3.1. Participant Recruitment and Demographic Data

Screening was conducted from 5 November 2021 to 15 December 2021, and 100 participants were recruited. All participants met the inclusion criteria. Therefore, they were all included in the study without any further exclusions. Table 1 shows the demographic characteristics of the study participants.

### 3.2. Comparison of Concordance in the MD Group

During the diagnosis of malposition in the MD group, the kappa coefficient was observed from −0.144 to 0.081, according to the evaluation items. Therefore, the concordance among the investigators was very low, corresponding with poor to slight according to the criteria by Landis and Koch [40] (Table 2). During the diagnosis of listhesis, the number of responses diagnosed with listhesis was extremely low for every rater. Paradoxically, even if the concordance was high when the responses were concentrated on a specific element, Fleiss’ kappa coefficient was low and statistically nonsignificant [41]. Therefore, only the response distribution for each evaluation factor by the investigators is described in Table S1.

### 3.3. Comparison of Concordance in the XE Group

With regard to the diagnosis of malposition in the XE group, the overall kappa coefficients ranged from 0.553 to 0.903. Therefore, the distribution was moderate to almost perfect according to the criteria proposed by Landis and Koch [40]. The concordance was the highest among all CMT spinal diagnostic methods used in this study. When comparing the evaluation items by segment, the concordance was relatively high for all evaluation items at L2–L4 levels (κ = 0.711–0.903). However, the kappa coefficients in the sagittal plane at L1 and L5 levels were 0.553 and 0.607, respectively, which were relatively low compared to other elements (Table 2).

In the diagnosis of listhesis, more than 98 elements were checked for the absence of malposition in all segments. Therefore, only the response distribution for each evaluation element is described in the supplementary file (Table S1).

### 3.4. Comparison of Concordance in the XN Group

With regard to diagnosis of malposition in the XN group, the overall kappa coefficients ranged from 0.102 to 0.625. According to the criteria proposed by Landis and Koch [40], the distribution varied from slight to substantial, which was lower than that of the XE group. Whereas concordance in the sagittal plane of the XE group was decreased in some segments, that of the XN group was decreased in all segments (κ = 0.012–0.19). The values corresponded to slight according to the criteria by Landis and Koch [40]. On the other hand, the concordance in the axial plane was the highest in all segments (Table 2).

With regard to the diagnosis of listhesis, many elements were determined as not having listhesis. Therefore, only the response distribution for each element is described in Table S1.

### 3.5. Comparison of Concordance in the AI Group

In the AI group, the kappa coefficients ranged from 0.193 to 0.803. According to the criteria proposed by Landis and Koch [40], the values ranged from slight to almost perfect. When examining each element, diagnosis concordance was higher than that without using the CMT-AI program in all elements except for malposition evaluation in the L4 level axial plane. However, similar to the XN group, the diagnostic concordance for malposition evaluation in the sagittal plane of L1–L4 levels was lower than that of other elements. In particular, the kappa coefficient at the L3 level was 0.193, which corresponded to slight according to the criteria proposed by Landis and Koch [40] (Table 2).

When evaluating listhesis, the statistical significance of the kappa coefficient decreased. However, more than 97 elements were determined as having no listhesis; the change was similar to that in the XE group. The data are presented in the supplementary file (Table S1).

### 3.6. Comparison of Concordance among CMT Diagnostic Methods

When analyzing the malposition diagnostic concordance among the diagnostic methods, the kappa coefficients ranged from 0.122 to 0.528, corresponding to slight to moderate level according to the criteria proposed by Landis and Koch [40]. Among various elements, the L1–L3 segment showed the highest concordance in the axial plane, and the L4-L5 segment showed the highest concordance in the coronal plane. Even in the comparison among diagnostic methods, the kappa coefficient in the sagittal plane was −0.043–0.116, showing the lowest concordance (Table 3).

In the analysis of listhesis, many elements were determined as having no listhesis. Therefore, only the response distribution for each element is described in Table S1.

When the concordance between the gold standard of CMT diagnosis (XE1) and the representatives of the groups (MD1, XN3, and AI3) was compared one on one, the concordance was the highest in AI3, followed by that in XN3 and MD1. MD1 had a lower concordance with XE1 than representatives of other groups. The kappa coefficient ranged from −0.088 to 0.212 in the presence of malposition. In addition, the kappa coefficient was negative in L1–L4 segments in the sagittal plane and the L4 segment in the axial plane. Comparing the concordance before and after CMT-AI program application, the kappa coefficient increased in 12 of 20 elements after AI program application. When the strength of agreement section was analyzed according to the criteria proposed by Landis and Koch [40], 19 of the 20 elements showed the same or an increased strength. When observed by individual elements, XN3 and AI3 had the lowest diagnostic concordance in the sagittal plane in most segments (Table 4).

## 4. Discussion

Previous studies on CMT diagnosis [31,35] had various limitations. First, the number of study participants was small, and there was only one diagnostic rater. Therefore, it was impossible to analyze the concordance among diagnoses based on the diagnostic modality. Also, the percentage of concordant diagnoses was used to compare the concordance; therefore, the probability of coincidence could not be excluded. Another study on XRD for CMT diagnosis [17] described methods for CMT diagnosis based on X-ray images by synthesizing the positional characteristics of the vertebrae and surrounding structures. However, this study was limited to methodological contents, and analysis of the diagnostic concordance between MD and XRD was not performed.

Our study overcame the limitations of previous studies on concordance evaluation for the current manual medical diagnosis and explored a rational and standard CMT diagnostic method in the following ways. First, in this study, CMT expert and non-expert groups were formed according to the criteria prepared in advance; MD and XRD, which are typically used in CMT spinal diagnosis, were performed according to SOPs; and concordance within and among the diagnosis groups was analyzed to identify a reasonable CMT diagnostic standard. Second, 100 clinical study participants were recruited based on previous studies, and the sample size was larger than that of previous similar studies. Finally, errors appearing as coincidence could be excluded using the kappa coefficient [39], a specialized tool for evaluating the reliability of a diagnosis expressed on a nominal scale.

The results of this study revealed very low concordance in MD within the group; further, concordance between the gold standard and representative of the MD group was low. Conversely, in the CMT expert group, the diagnostic concordance of XRD was high, corresponding to moderate to almost perfect according to the criteria proposed by Landis and Koch [40]. Therefore, XRD may be a reasonable and standard CMT diagnostic method. Although it was possible to determine the presence and approximate direction of malposition and listhesis using MD, it was difficult to determine the exact direction and extent of malposition and listhesis. Furthermore, an unnecessary load generated on the spine when the spine is not aligned can result in creep deformation of the ligaments [42], and the segment can easily get damaged with less than normal force [43]. In particular, when comparing the force applied in the high-velocity low-amplitude technique with a force that can damage the spine [44,45], it is possible to damage the structures around the spine if the force is applied in the wrong segment or direction. Therefore, it may be more effective to perform XRD before CMT. In addition, efforts have been made to apply imaging tests to enhance the objectivity of the diagnosis not only in CMT but also in other manual therapies [15,16]. In modern medicine, X-ray examination is commonly used to monitor treatment effects or to determine disease progression [46], and it is effective in identifying underlying diseases of the spine and contraindications of CMT [15]. Although it has a risk of radiation exposure, the effect is negligible [47]. Plus, it is a convenient and time-saving method that can identify osseous tissue, its cost is low, and it has little variation between investigators [48,49]. Therefore, XRD may be a practical diagnostic method for CMT and other manual therapies.

According to this study, the inter-rater concordance of the MD group ranged from poor to slight according to the criteria by Landis and Koch [40], relatively lower than that of other groups. Although the CMT experts have undergone a similar training process and are experienced KMDs, individual deviations could occur because MD relies on the senses. This phenomenon also occurs in other manual medicine. For instance, the inter-rater concordance in MD was low even among experts who received the same residency training for neuromusculoskeletal medicine and osteopathy, which increased slightly after the implementation of consensus training on diagnostic methods [50]. In addition, the concordance may have been affected by the methodological limitations of this study. Three raters diagnosed the participants sequentially. Similar to the diagnostic method, CMT has a joint mobilizing technique that moves the patient’s body by palpating bony landmarks [8], which may exert a therapeutic effect during the diagnostic process. Osteopathy also uses a diagnostic method to track movements by palpating bony landmarks, and therapeutic effects can occur in the process [32]. Furthermore, no vertebral joints were diagnosed during the screening process. Therefore, it cannot be concluded that the vertebral joints are not a condition that can be easily changed because the participant’s vertebral joint is unstable [51], suggesting that the malposition may have been corrected or changed during the diagnostic process. Thus, the concordance of the MD group may be increased by supplementing the limitations when conducting additional studies in the future.

This study showed decreased diagnostic concordance between the gold standard and MD group. In particular, the kappa coefficients were negative in the sagittal plane of L1–L4 segments and axial plane of L4 segment when evaluating the malposition. MD is a method to check the spine’s mobility, and XRD is a method to check malalignment. Diagnosis may differ because of differences in biomechanical perspectives of the two diagnostic methods. Other manual therapies, which treat the spine after diagnosis, mainly diagnose structures from a static perspective in the initial stage. However, restriction can occur even if the alignment is not incorrect, and abnormal joint alignment does not necessarily indicate joint dysfunction or limited mobility [52]. In CMT, malposition is diagnosed and treated; in addition, the lesion site is diagnosed from static and dynamic perspectives considering biomechanical aspects [8]. Moreover, mobility problems of the lumbar spine are found in most patients with low back pain, and hypermobility in most cases occurs as compensation for low mobility [53]. Therefore, it is necessary to consider both MD and XRD in diagnosis and treatment.

With regard to XRD, the concordance in the expert group was high, but not in the non-expert group. Among them, the concordance of diagnosis for malposition in the sagittal plane, in particular, was not high, which is thought to be related to the current situation of a lack of consensus on malposition in the sagittal plane. Currently, CMT spinal diagnosis is based on the position difference of the upper vertebral body relative to the lower vertebral body, but quantitative criteria are not presented. Moreover, Lateral bending and rotation can assume an accurate neutral position. In contrast, this is not true for flexion and extension because of physiological lordosis of the lumbar spine [54]. Therefore, the inter-rater concordance for malposition diagnosis in the sagittal plane was low. This trend was prominent in the non-expert group, whose members were unfamiliar with the CMT diagnosis. Therefore, quantitative criteria for the extent of flexion and extension malposition relative to the sagittal plane should be established to increase the accuracy of XRD and concordance among diagnoses.

This study is the first to apply the previously reported CMT-AI diagnosis program to clinical practice. With the CMT-AI diagnostic program, the intragroup diagnostic concordance increased compared to that before program application, even in the CMT non-expert group. In addition, the result of maintaining or increasing the strength of agreement was also observed in comparing diagnostic concordance with CMT experts. Therefore, a more rational diagnosis can be made by compensating the immaturity of diagnosis observed in CMT non-experts through the CMT-AI program. In relation to diagnosis in Korean traditional medicine and manual medicine, this study expands on the existing research [28] that focused only on detecting the location of the vertebral body with the AI program. Therefore, it is meaningful that we could examine the possibility of applying the program to clinical practice through our finding that the AI program is helpful for CMT spinal diagnosis.

The study has some limitations. First, there were problems related to lumbar instability and diagnostic process of the study participants, as mentioned earlier. In addition, the prevalence of listhesis is 19.1% in men, 25% in women aged >65 years [55], and 8.7% in all patients with low back pain [56]. In this study, the average age of participants was 32.5 ± 11.3 years, and recruited participants included healthy subjects. Therefore, only 0–2 cases of listhesis per segment were diagnosed according to the gold standard. When the diagnosis frequency was extremely low, Fleiss’ kappa coefficient was low even when the concordance was high [41]. Therefore, statistical analysis for listhesis could not be performed. In the future, a diagnostic concordance study for listhesis needs to be conducted by recruiting only patients diagnosed with listhesis.

Furthermore, although this study confirmed reliability by comparing intra- and inter-group concordance of diagnostic methods, we did not confirm the validity of the diagnostic method for individual raters. Clinical usefulness requires comprehensive consideration of reliability, validity, responsiveness, and utility [57]. However, it was difficult to ascertain the validity of CMT spinal diagnosis because there is currently no quantitative standard for diagnosis. The diagnosis of malposition in the sagittal plane also seems to have been influenced. Therefore, it is necessary to prepare accurate diagnostic criteria for CMT spinal diagnosis in the future, and additional studies are needed on the validity of each diagnostic method.

## 5. Conclusions

Through this study, it was found that X-ray image-based diagnosis, which has higher intragroup concordance than manual diagnosis, was a more reasonable Chuna manual therapy (CMT) spinal diagnostic method. In addition, when the CMT-artificial intelligence (AI) program was used, the diagnostic concordance within the CMT non-expert group and diagnostic concordance with the gold standard increased, suggesting that the clinical application of the CMT spinal diagnostic method through the AI program is highly recommended. There were some limitations in the study design, such as problems related to lumbar instability and the absence of a quantitative standard for the diagnosis of malposition in the sagittal plane. However, this study was conducted with a relatively large sample size and is the first study to analyze the concordance within and among different diagnostic methods, including a diagnostic method utilizing an AI program. We believe that it may serve as the basis for randomized controlled trials of larger scale, and the development of CMT diagnostic methods in the future.

## Figures and Tables

**Table 1:** Demographic characteristics of participants.

Item		(*n* = 100)
Age (years; mean ± SD)		32.5 ± 11.3
Sex	Male [*n* (%)]	36 (36)
	Female [*n* (%)]	64 (64)
Height (cm; mean ± SD)		165.0 ± 8.2
Weight (kg; mean ± SD)		62.8 ± 10.8
BMI (kg/m^2^; mean ± SD)		22.9 ± 2.7
BP	Systolic (mmHg; mean ± SD)	120.6 ± 10.5
	Diastolic (mmHg; mean ± SD)	75.6 ± 8.5
HR (bpm; mean ± SD)		78.9 ± 10.6
BT (°C; mean ± SD)		36.7 ± 0.3
LBP (NRS; mean ± SD)		2.4 ± 1.6

BMI, body mass index; BP, blood pressure; bpm, beats per minute; BT, body temperature; HR, heart rate; LBP, Lower back pain; NRS, numeric rating scale; SD, standard deviation.

**Table 2:** Comparison of diagnostic concordance for lumbar spine malposition within diagnostic groups.

		MD Group			XE Group			XN Group			AI Group		
Level	Item *	Kappa	Z	*p*-Value	Kappa	Z	*p*-Value	Kappa	Z	*p*-Value	Kappa	Z	*p*-Value
L1	Mal ^†^	−0.104	−1.8	0.073	0.587	10.2	0.000	0.295	5.1	0.000	0.465	8.1	0.000
	Sag ^‡^	−0.064	−1.4	0.152	0.553	10.1	0.000	0.012	0.2	0.808	0.470	8.6	0.000
	Axi ^§^	−0.042	−1.0	0.324	0.826	19.5	0.000	0.625	14.7	0.000	0.803	19.2	0.000
	Cor ^∥^	−0.078	−1.8	0.067	0.840	19.4	0.000	0.432	9.9	0.000	0.647	15.2	0.000
L2	Mal ^†^	0.018	0.3	0.751	0.790	13.7	0.000	0.428	7.4	0.000	0.476	8.2	0.000
	Sag ^‡^	−0.004	−0.1	0.927	0.860	17.2	0.000	0.151	3.4	0.001	0.662	13.4	0.000
	Axi ^§^	0.001	0.0	0.986	0.876	20.8	0.000	0.597	14.1	0.000	0.718	17.2	0.000
	Cor ^∥^	−0.037	−0.9	0.369	0.834	19.8	0.000	0.561	13	0.000	0.711	17.3	0.000
L3	Mal ^†^	−0.081	−1.4	0.162	0.747	12.9	0.000	0.428	7.4	0.000	0.492	8.5	0.000
	Sag ^‡^	0.081	1.8	0.066	0.769	16.9	0.000	0.086	1.8	0.066	0.193	4.2	0.000
	Axi ^§^	−0.059	−1.4	0.173	0.842	19.8	0.000	0.598	14.1	0.000	0.753	17.7	0.000
	Cor ^∥^	−0.079	−1.9	0.061	0.751	17.0	0.000	0.394	8.8	0.000	0.670	15.7	0.000
L4	Mal ^†^	0.006	0.1	0.919	0.715	12.4	0.000	0.473	8.2	0.000	0.485	8.4	0.000
	Sag ^‡^	−0.007	−0.2	0.866	0.721	16.1	0.000	0.190	4.0	0.000	0.312	7.0	0.000
	Axi ^§^	−0.052	−1.2	0.222	0.903	20.6	0.000	0.594	13.7	0.000	0.588	13.5	0.000
	Cor ^∥^	−0.017	−0.4	0.683	0.711	16.3	0.000	0.349	8.0	0.000	0.636	14.9	0.000
L5	Mal ^†^	−0.097	−1.7	0.092	0.629	10.9	0.000	0.134	2.3	0.020	0.572	9.9	0.000
	Sag ^‡^	−0.056	−1.3	0.180	0.607	13.6	0.000	0.061	1.3	0.185	0.477	10.1	0.000
	Axi ^§^	−0.111	−2.6	0.008	0.861	18.7	0.000	0.220	4.9	0.000	0.352	8.0	0.000
	Cor ^∥^	−0.144	−3.4	0.001	0.694	15.1	0.000	0.102	2.2	0.030	0.543	12.3	0.000

* Item: All investigators of each group checked the presence of malposition ^†^; subsequently, they evaluated malposition of the sagittal ^‡^ (flexion, extension, neutral), axial ^§^ (right and left rotation, neutral), and coronal planes ^∥^ (right and left lateral bending). Thereafter, the concordance within groups was compared. AI, X-ray image-based diagnosis using artificial intelligence program by non-experts; Axi, axial plane; Cor, coronal plane; Mal, presence of malposition; MD, manual diagnosis by experts; Sag, sagittal plane; XE; X-ray image-based diagnosis by experts; XN; X-ray image-based diagnosis by non-experts.

**Table 3:** Comparison of concordance among CMT diagnostic methods.

Level	Item *	Kappa	Z	*p*-Value
L1	Mal ^†^	0.286	7.0	0.000
	Sag ^‡^	0.116	3.2	0.001
	Axi ^§^	0.493	16.5	0.000
	Cor ^∥^	0.396	13.1	0.000
L2	Mal ^†^	0.262	6.4	0.000
	Sag ^‡^	0.103	3.3	0.001
	Axi ^§^	0.414	14	0.000
	Cor ^∥^	0.406	13.8	0.000
L3	Mal ^†^	0.222	5.4	0.000
	Sag ^‡^	0.041	1.3	0.194
	Axi ^§^	0.528	17.6	0.000
	Cor ^∥^	0.250	8.1	0.000
L4	Mal ^†^	0.173	4.2	0.000
	Sag ^‡^	−0.026	0.8	0.421
	Axi ^§^	0.238	7.9	0.000
	Cor ^∥^	0.245	8.1	0.000
L5	Mal ^†^	0.142	3.5	0.000
	Sag ^‡^	−0.043	−1.4	0.163
	Axi ^§^	0.122	3.9	0.000
	Cor ^∥^	0.154	5.0	0.000

* Item: All investigators assessed the presence of malposition ^†^; subsequently, they evaluated malposition of the sagittal ^‡^ (flexion, extension, neutral), axial ^§^ (right and left rotation, neutral), and coronal planes ^∥^ (right and left lateral bending). Thereafter, the concordance among groups was compared. Axi, axial plane; Cor, coronal plane; Mal, presence of malposition; Sag, sagittal plane.

**Table 4:** Comparison of diagnostic concordance among the gold standard and diagnosis of representative for each diagnostic group.

Level		L1				L2				L3				L4				L5			
Item *		Mal ^†^	Sag ^‡^	Axi ^§^	Cor ^∥^	Mal ^†^	Sag ^‡^	Axi ^§^	Cor ^∥^	Mal ^†^	Sag ^‡^	Axi ^§^	Cor ^∥^	Mal ^†^	Sag ^‡^	Axi ^§^	Cor ^∥^	Mal ^†^	Sag ^‡^	Axi ^§^	Cor ^∥^
CI	MD1	0.212 (0.025)	−0.053 (0.427)	0.411 (0.000)	0.359 (0.000)	0.079 (0.000)	−0.053 (0.390)	0.202 (0.003)	0.255 (0.000)	0.042 (0.677)	−0.073 (0.216)	0.231 (0.001)	0.193 (0.007)	−0.088 (0.368)	−0.053 (0.314)	−0.100 (0.115)	0.057 (0.393)	0.071 (0.307)	0.036 (0.370)	0.085 (0.005)	0.159 (0.000)
		++	-	+++	++	+	-	++	++	+	-	++	+	-	-	-	+	+	+	+	+
	XN3	0.541 (0.000)	0.149 (0.003)	0.680 (0.000)	0.589 (0.000)	0.498 (0.000)	0.310 (0.000)	0.641 (0.000)	0.559 (0.000)	0.602 (0.000)	0.340 (0.000)	0.793 (0.000)	0.416 (0.000)	0.529 (0.000)	0.064 (0.224)	0.782 (0.000)	0.551 (0.000)	0.318 (0.000)	0.093 (0.002)	0.522 (0.000)	0.311 (0.000)
		+++	+	++++	+++	+++	++	++++	+++	++++	++	++++	+++	+++	+	++++	+++	++	+	+++	++
	AI3	0.442 (0.000)	0.553 (0.000)	0.667 (0.000)	0.656 (0.000)	0.508 (0.000)	0.668 (0.000)	0.703 (0.000)	0.725 (0.000)	0.430 (0.000)	0.310 (0.000)	0.783 (0.000)	0.558 (0.000)	0.495 (0.000)	0.259 (0.001)	0.692 (0.000)	0.611 (0.000)	0.506 (0.000)	0.457 (0.000)	0.446 (0.000)	0.655 (0.000)
		+++	+++	++++	++++	+++	++++	++++	++++	+++	++	++++	+++	+++	++	++++	++++	+++	+++	+++	++++

(1) Data: Cohen’s kappa coefficient, (*p*-value), strength of agreement (-, +, ++, +++, ++++, +++++). (2) Strength of agreement: -, Poor level of concordance by Landis and Koch [40] [κ > 0.000]; +, Slight (κ = 0.000–0.200); ++, Fair (κ = 0.200–0.400); +++, Moderate (κ = 0.400–0.600); ++++, Substantial (κ = 0.600–0.800); +++++, Almost perfect (κ = 0.800–1.000). * Item: All investigators assessed for the presence of malposition ^†^; subsequently, they evaluated the malposition of the sagittal ^‡^ (flexion, extension, neutral), axial ^§^ (right and left rotation, neutral), and coronal planes ^∥^ (right and left lateral bending). Thereafter, concordance among investigators was compared. AI3, concordance between the gold standard and diagnosis by the AI group representative; Axi, axial plane; Cor, coronal plane; CI, comparative investigator; Mal, presence of malposition; MD1, concordance between the gold standard and diagnosis by the MD group representative; Sag, sagittal plane; XN3, concordance between the gold standard and diagnosis by the XN group representative.

## Data Availability

The data presented in this study are available upon reasonable request.

## Supplementary Materials

The following supporting information can be downloaded at: https://www.mdpi.com/article/10.3390/diagnostics12112732/s1. Figure S1, A detailed description of the malposition diagnosis through the manual method with the transverse process palpated on the left; Table S1, Number of diagnoses by detailed item in listhesis diagnosis; Table S2, Number of diagnoses by detailed item in malposition diagnosis.
