# Supplementary material for: Comparison of Concordance between Chuna Manual Therapy Diagnostic Methods (Palpation, X-ray, Artificial Intelligence Program) in Lumbar Spine: An Exploratory, Cross-Sectional Clinical Study
Source: Diagnostics (Basel). 2022 Nov 8;12(11):2732. doi: 10.3390/diagnostics12112732 (PMC9689192; doi:10.3390/diagnostics12112732)
Supplement: Supplementary file 1 [file diagnostics-12-02732-s001.zip › diagnostics-1978275-supplementary.pdf]

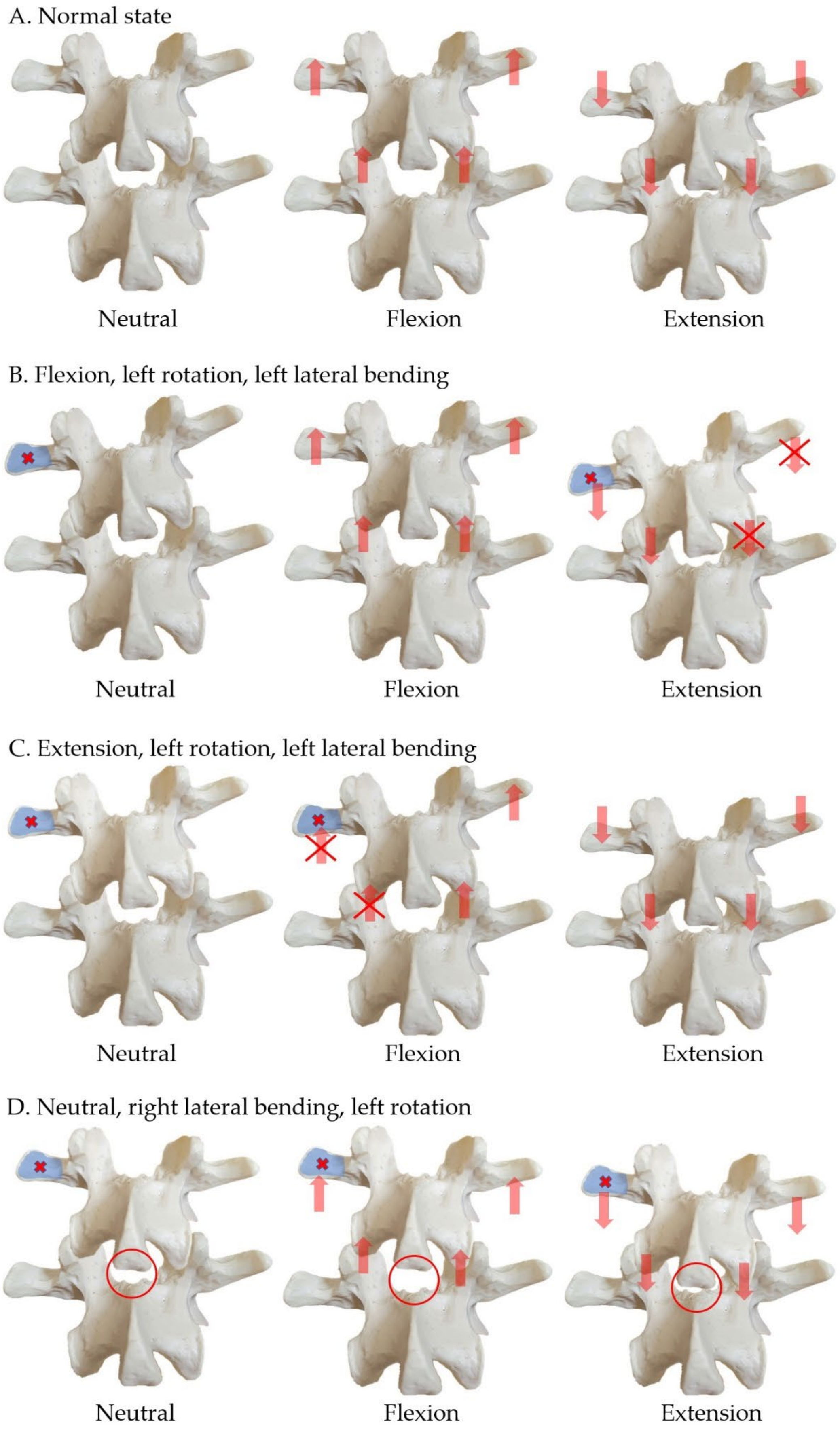


**Figure S1**. A detailed description of the malposition diagnosis through the manual method with the transverse process palpated on the left[†]. A, normal; B, FRS$_{L}$; C, ERS$_{L}$; D, NR$_{R}$S$_{L}$.

[†]If the transverse process is palpated on the right, the figure above should be considered in reverse.

Cross marks on the transverse process, relatively more palpable transverse processes with limited movement; F, flexion; E, extension; S, side bending (lateral bending); R, right side; L, left side.

**Table S1.** Number of diagnoses by detailed elements in listhesis diagnosis

| | | | MD group | | | XE group | | | XN group | | | AI group | | |
|---|---|---|---|---|---|---|---|---|---|---|---|---|---|---|
| Level | Item* | Var† | MD1 | MD2 | MD3 | XE1 | XE2 | XE3 | XN1 | XN2 | XN3 | AI1 | AI2 | AI3 |
| L1 | Lis‡ | Yes (n) | 0 | 5 | 4 | 0 | 0 | 0 | 0 | 2 | 0 | 0 | 0 | 0 |
| | | No (n) | 100 | 95 | 96 | 100 | 100 | 100 | 100 | 98 | 100 | 100 | 100 | 100 |
| | Cor§ | Lt. (n) | 0 | 4 | 0 | 0 | 0 | 0 | 0 | 0 | 0 | 0 | 0 | 0 |
| | | Rt. (n) | 0 | 1 | 0 | 0 | 0 | 0 | 0 | 0 | 0 | 0 | 0 | 0 |
| | | N (n) | 100 | 95 | 100 | 100 | 100 | 100 | 100 | 100 | 100 | 100 | 100 | 100 |
| | Sag‖ | Ant. (n) | 0 | 0 | 2 | 0 | 0 | 0 | 0 | 0 | 0 | 0 | 0 | 0 |
| | | Ret. (n) | 0 | 0 | 2 | 0 | 0 | 0 | 0 | 2 | 0 | 0 | 0 | 0 |
| | | N (n) | 100 | 100 | 96 | 100 | 100 | 100 | 100 | 98 | 100 | 100 | 100 | 100 |
| L2 | Lis‡ | Yes (n) | 1 | 11 | 16 | 2 | 2 | 2 | 7 | 12 | 0 | 0 | 2 | 1 |
| | | No (n) | 99 | 89 | 84 | 98 | 98 | 98 | 93 | 88 | 100 | 100 | 98 | 99 |
| | Cor§ | Lt. (n) | 0 | 6 | 1 | 0 | 0 | 1 | 1 | 1 | 0 | 0 | 0 | 0 |
| | | Rt. (n) | 1 | 3 | 1 | 0 | 0 | 0 | 1 | 0 | 0 | 0 | 0 | 0 |
| | | N (n) | 99 | 91 | 98 | 100 | 100 | 99 | 98 | 99 | 100 | 100 | 100 | 100 |
| | Sag‖ | Ant. (n) | 0 | 1 | 5 | 0 | 0 | 0 | 0 | 0 | 0 | 0 | 0 | 0 |
| | | Ret. (n) | 0 | 2 | 10 | 2 | 2 | 2 | 6 | 12 | 0 | 0 | 2 | 1 |
| | | N (n) | 100 | 97 | 85 | 98 | 98 | 98 | 94 | 88 | 100 | 100 | 98 | 99 |
| L3 | Lis‡ | Yes (n) | 1 | 14 | 9 | 0 | 1 | 0 | 16 | 10 | 0 | 1 | 0 | 1 |
| | | No (n) | 99 | 86 | 91 | 100 | 99 | 100 | 84 | 90 | 100 | 99 | 100 | 99 |
| | Cor§ | Lt. (n) | 0 | 5 | 1 | 0 | 0 | 0 | 1 | 1 | 0 | 0 | 0 | 1 |
| | | Rt. (n) | 0 | 1 | 0 | 0 | 0 | 0 | 1 | 0 | 0 | 1 | 0 | 0 |
| | | N (n) | 100 | 94 | 99 | 100 | 100 | 100 | 98 | 99 | 100 | 99 | 100 | 99 |
| | Sag‖ | Ant. (n) | 1 | 7 | 4 | 0 | 0 | 0 | 1 | 0 | 0 | 0 | 0 | 0 |
| | | Ret. (n) | 0 | 1 | 4 | 0 | 1 | 0 | 13 | 9 | 0 | 0 | 0 | 0 |
| | | N (n) | 99 | 92 | 92 | 100 | 99 | 100 | 86 | 91 | 100 | 100 | 100 | 100 |
| L4 | Lis‡ | Yes (n) | 0 | 2 | 7 | 1 | 2 | 2 | 15 | 12 | 0 | 2 | 3 | 3 |
| | | No (n) | 100 | 98 | 93 | 99 | 98 | 98 | 85 | 88 | 100 | 98 | 97 | 97 |
| | Cor§ | Lt. (n) | 0 | 1 | 0 | 1 | 1 | 1 | 1 | 3 | 0 | 1 | 1 | 1 |
| | | Rt. (n) | 0 | 0 | 0 | 0 | 0 | 0 | 0 | 0 | 0 | 0 | 0 | 0 |
| | | N (n) | 100 | 99 | 100 | 99 | 99 | 99 | 99 | 97 | 100 | 99 | 99 | 99 |
| | Sag‖ | Ant. (n) | 0 | 0 | 3 | 0 | 0 | 0 | 2 | 0 | 0 | 0 | 0 | 0 |
| | | Ret. (n) | 0 | 1 | 4 | 0 | 1 | 1 | 11 | 10 | 0 | 1 | 2 | 2 |
| | | N (n) | 100 | 99 | 93 | 100 | 99 | 99 | 87 | 90 | 100 | 99 | 98 | 98 |
| L5 | Lis‡ | Yes (n) | 0 | 0 | 4 | 2 | 1 | 0 | 1 | 2 | 0 | 2 | 2 | 3 |
| | | No (n) | 100 | 100 | 96 | 98 | 99 | 100 | 99 | 98 | 100 | 98 | 98 | 97 |
| | Cor§ | Lt. (n) | 0 | 0 | 0 | 0 | 0 | 0 | 0 | 0 | 0 | 0 | 0 | 0 |
| | | Rt. (n) | 0 | 0 | 0 | 0 | 0 | 0 | 0 | 0 | 0 | 0 | 0 | 0 |
| | | N (n) | 100 | 100 | 100 | 100 | 100 | 100 | 100 | 100 | 100 | 100 | 100 | 100 |
| | Sag‖ | Ant. (n) | 0 | 0 | 0 | 0 | 1 | 0 | 0 | 1 | 0 | 0 | 0 | 0 |
| | | Ret. (n) | 0 | 0 | 4 | 2 | 0 | 0 | 1 | 1 | 0 | 2 | 2 | 3 |
| | | N (n) | 100 | 100 | 96 | 98 | 99 | 100 | 99 | 98 | 100 | 98 | 98 | 97 |

*Element: All investigators determined the presence of listhesis‡; subsequently, they evaluated listhesis of the coronal§ (right and left laterolisthesis, neutral) and sagittal planes‖ (anterolisthesis, retrolisthesis, neutral).

†Var: (variables) The number of diagnoses made by each investigator is indicated.

AI, X-ray based diagnosis using artificial intelligence program by non-experts; Ant., anterolisthesis; Cor, coronal plane; Lis, presence of listhesis, Lt., left; N, neutral; Ret., retrolisthesis; Rt., right; Sag, sagittal plane; MD, manual diagnosis by experts; XE; X-ray-based diagnosis by experts; XN; X-ray-based diagnosis by non-experts.

**Table S2.** Number of diagnoses by detailed elements in malposition diagnosis

| | | | MD group | | | XE group | | | XN group | | | AI group | | |
|---|---|---|---|---|---|---|---|---|---|---|---|---|---|---|
| Level | Item* | Var† | MD1 | MD2 | MD3 | XE1 | XE2 | XE3 | XN1 | XN2 | XN3 | AI1 | AI2 | AI3 |
| | Mal‡ | Yes (n) | 47 | 11 | 60 | 63 | 74 | 51 | 64 | 80 | 41 | 82 | 70 | 77 |
| | | No (n) | 53 | 89 | 40 | 37 | 26 | 49 | 36 | 20 | 59 | 18 | 30 | 23 |
| | Sag§ | Flex. (n) | 11 | 4 | 33 | 37 | 51 | 22 | 17 | 52 | 5 | 52 | 37 | 31 |
| | | Ext. (n) | 12 | 4 | 14 | 3 | 0 | 2 | 15 | 0 | 0 | 5 | 1 | 0 |
| | | N (n) | 77 | 92 | 53 | 60 | 49 | 76 | 68 | 48 | 95 | 43 | 62 | 69 |
| L1 | Axi‖ | Rt. (n) | 25 | 4 | 20 | 21 | 24 | 16 | 22 | 27 | 16 | 20 | 25 | 24 |
| | | Lt. (n) | 22 | 4 | 35 | 21 | 21 | 23 | 22 | 19 | 14 | 23 | 24 | 25 |
| | | N (n) | 53 | 92 | 45 | 58 | 55 | 61 | 56 | 54 | 70 | 57 | 51 | 51 |
| | Cor** | Rt. (n) | 21 | 5 | 29 | 20 | 17 | 19 | 29 | 23 | 15 | 33 | 21 | 34 |
| | | Lt. (n) | 26 | 2 | 26 | 16 | 17 | 11 | 20 | 14 | 7 | 22 | 14 | 18 |
| | | N (n) | 53 | 93 | 45 | 64 | 66 | 70 | 51 | 63 | 78 | 45 | 65 | 48 |
| | Mal‡ | Yes (n) | 67 | 31 | 66 | 63 | 59 | 60 | 64 | 65 | 43 | 74 | 67 | 78 |
| | | No (n) | 33 | 69 | 34 | 37 | 41 | 40 | 36 | 35 | 57 | 26 | 33 | 22 |
| | Sag§ | Flex. (n) | 16 | 11 | 34 | 15 | 12 | 14 | 8 | 13 | 3 | 19 | 13 | 10 |
| | | Ext. (n) | 27 | 11 | 8 | 4 | 2 | 3 | 21 | 3 | 1 | 6 | 2 | 2 |
| | | N (n) | 57 | 78 | 58 | 81 | 86 | 83 | 71 | 84 | 96 | 75 | 85 | 88 |
| L2 | Axi‖ | Rt. (n) | 30 | 13 | 17 | 22 | 26 | 19 | 22 | 30 | 17 | 20 | 28 | 25 |
| | | Lt. (n) | 36 | 12 | 45 | 23 | 21 | 22 | 23 | 23 | 16 | 24 | 24 | 23 |
| | | N (n) | 34 | 75 | 38 | 55 | 53 | 59 | 55 | 47 | 67 | 56 | 48 | 52 |
| | Cor** | Rt. (n) | 26 | 16 | 35 | 30 | 19 | 27 | 33 | 29 | 18 | 36 | 31 | 35 |
| | | Lt. (n) | 40 | 7 | 27 | 21 | 21 | 20 | 17 | 21 | 8 | 29 | 22 | 28 |
| | | N (n) | 34 | 77 | 38 | 49 | 60 | 53 | 50 | 50 | 74 | 35 | 47 | 37 |
| | Mal‡ | Yes (n) | 48 | 34 | 72 | 52 | 51 | 48 | 65 | 63 | 44 | 64 | 57 | 72 |
| | | No (n) | 52 | 66 | 28 | 48 | 49 | 52 | 35 | 37 | 56 | 36 | 43 | 28 |
| | Sag§ | Flex. (n) | 13 | 15 | 34 | 5 | 5 | 7 | 7 | 2 | 3 | 11 | 3 | 3 |
| | | Ext. (n) | 16 | 12 | 9 | 3 | 5 | 3 | 19 | 8 | 0 | 0 | 5 | 6 |
| | | N (n) | 71 | 73 | 57 | 92 | 90 | 90 | 74 | 90 | 97 | 89 | 92 | 91 |
| L3 | Axi‖ | Rt. (n) | 24 | 9 | 14 | 20 | 23 | 18 | 20 | 31 | 18 | 16 | 20 | 20 |
| | | Lt. (n) | 23 | 17 | 53 | 19 | 17 | 19 | 21 | 22 | 16 | 16 | 22 | 19 |
| | | N (n) | 53 | 74 | 33 | 61 | 60 | 63 | 59 | 47 | 66 | 68 | 58 | 61 |
| | Cor** | Rt. (n) | 24 | 14 | 39 | 23 | 14 | 18 | 32 | 26 | 14 | 28 | 24 | 27 |
| | | Lt. (n) | 23 | 12 | 28 | 11 | 12 | 10 | 16 | 16 | 2 | 20 | 13 | 20 |
| | | N (n) | 53 | 74 | 33 | 66 | 74 | 72 | 52 | 58 | 84 | 52 | 63 | 53 |
| | Mal‡ | Yes (n) | 57 | 40 | 66 | 46 | 43 | 42 | 45 | 53 | 35 | 52 | 49 | 68 |
| | | No (n) | 43 | 60 | 34 | 54 | 57 | 58 | 55 | 47 | 65 | 48 | 51 | 32 |
| | Sag§ | Flex. (n) | 13 | 16 | 28 | 9 | 8 | 11 | 4 | 5 | 1 | 12 | 7 | 6 |
| | | Ext. (n) | 43 | 15 | 13 | 8 | 8 | 5 | 16 | 9 | 2 | 1 | 9 | 11 |
| | | N (n) | 44 | 69 | 59 | 83 | 84 | 84 | 80 | 86 | 97 | 87 | 84 | 83 |
| L4 | Axi‖ | Rt. (n) | 30 | 15 | 11 | 12 | 12 | 11 | 12 | 23 | 13 | 8 | 19 | 20 |
| | | Lt. (n) | 23 | 15 | 49 | 13 | 13 | 16 | 15 | 15 | 13 | 8 | 14 | 18 |
| | | N (n) | 47 | 67 | 40 | 75 | 75 | 73 | 73 | 62 | 74 | 84 | 67 | 62 |
| | Cor** | Rt. (n) | 32 | 19 | 34 | 15 | 13 | 10 | 13 | 17 | 10 | 19 | 16 | 17 |
| | | Lt. (n) | 21 | 13 | 26 | 16 | 13 | 16 | 24 | 18 | 7 | 24 | 18 | 26 |
| | | N (n) | 47 | 68 | 40 | 69 | 74 | 74 | 63 | 65 | 83 | 57 | 66 | 57 |
| L5 | Mal‡ | Yes (n) | 80 | 41 | 38 | 40 | 38 | 24 | 9 | 54 | 20 | 58 | 48 | 53 |

| | | | | | | | | | | | | | |
|---|---|---|---|---|---|---|---|---|---|---|---|---|---|
| | No (n) | 20 | 59 | 62 | 60 | 62 | 76 | 91 | 46 | 80 | 42 | 52 | 47 |
| Sag[§] | Flex. (n) | 39 | 14 | 13 | 11 | 11 | 4 | 1 | 14 | 0 | 22 | 9 | 15 |
| | Ext. (n) | 40 | 25 | 16 | 7 | 10 | 3 | 1 | 8 | 1 | 1 | 9 | 6 |
| | N (n) | 21 | 61 | 71 | 82 | 79 | 93 | 98 | 78 | 99 | 77 | 82 | 79 |
| Axi[‖] | Rt. (n) | 60 | 12 | 1 | 4 | 6 | 4 | 0 | 21 | 8 | 1 | 16 | 19 |
| | Lt. (n) | 19 | 7 | 34 | 9 | 9 | 8 | 3 | 10 | 7 | 5 | 14 | 14 |
| | N (n) | 21 | 81 | 65 | 87 | 85 | 88 | 97 | 69 | 85 | 94 | 70 | 67 |
| Cor[**] | Rt. (n) | 62 | 11 | 10 | 10 | 5 | 5 | 2 | 8 | 2 | 17 | 8 | 11 |
| | Lt. (n) | 18 | 7 | 25 | 17 | 16 | 12 | 4 | 22 | 4 | 26 | 21 | 17 |
| | N (n) | 20 | 82 | 65 | 73 | 79 | 83 | 94 | 70 | 94 | 57 | 71 | 72 |

[*]Element: All investigators assessed the presence of malposition[‡]; subsequently, they evaluated malposition of the sagittal[§](flexion, extension, neutral), axial[‖](right and left rotation, neutral), and coronal planes[**](right and left lateral bending).

[†]Var: (variables) The number of diagnoses performed by each investigator is indicated.

AI, X-ray-based diagnosis using artificial intelligence program by non-experts; Axi, axial plane; Cor, coronal plane; Lt., left; Mal, presence of malposition; MD, manual diagnosis by experts; N, neutral; Rt., right; Sag, sagittal plane; XE, X-ray-based diagnosis by experts; XN, X-ray-based diagnosis by non-experts
